# 

**Published:** 1890-06

**Authors:** 


### American Dental Association.

The thirteenth annual session of the American Dental Association will be held at Excelsior Springs, Missouri, commencing Tuesday, August 5th, 1890, at 10 o'clock A. M.

GEO. H. CUSHING, Rec. Sec., Am. Dent. Ass.

### Association of Dental College Faculties.

The annual meeting of this body will be held at Excelsior Springs, Mo., Tuesday, August 5th, 1890.

It is greatly to be desired that all the colleges in the country having membership in this body will be represented, as there will be important business that ought to receive due consideration, with reference to properly carrying out the resolution adopted at the last annual meeting in regard to extending the number of terms for graduation.

This resolution involves one of the greatest steps that has ever been taken in respect to dental education. It is very desirable that in arranging for the new scheme wisdom should be exercised, and the utmost harmony secured.

It is to be hoped that all other dental colleges in the country who approve of a good standard of dental education will ally themselves and become co-operative with this organization. We trust that the time has now arrived when the aim and effort of every dental college will be to accomplish the best work rather than to secure the largest number of students, and that more care and discrimination will be exercised in the future in regard to admitting students to their classes.

### National Association of Dental Examiners.

The annual meeting of this body will be held at Excelsior Springs, August 5th, 1890, and it is to be hoped that there will be a full representation from all the examining boards of the different States that have laws regulating the practice of dentistry.

This is a board upon which very much depends as to the educational interests of dentistry. Great discretion and wisdom should characterize its proceedings. This is emphasized when it is remembered that it possesses more power than any other organization in the dental profession.

Its work in the past has been of inestimable value, and very few, if any, mistakes have been made by it. As it goes on, its responsibility increases in proportion to the increase of its membership.

When this body was organized comparatively few States had laws regulating the practice of dentistry; now nearly all the States of the Union have such laws, and each one has its board of dental examiners, varying somewhat in the work committed to their charge, because mainly of variety in the laws of the different States. There is no special discord existing between the laws of the different states, but the requirements of some are much in advance of those of others.

The efforts of this association of boards should be directed to securing uniformity in these laws so far as possible, and in the main there will be no difficulty in securing uniformity in all the material points, and it is greatly to be hoped that this body will use its best efforts in securing this end.

### Georgia State Dental Society.

### Obituary.

Died, at Compton Creek, Fayette County, Ohio, April 18th, 1890, of heart disease, Dr. Luman Birch Welsch, D.D.S., of

### CONTENTS.

COMMUNICATIONS.

Evolution in Dentistry .... 265
Diseases of the Antrum .... 269

SELECTIONS.

A Study of the Relationship between the Eruption of the Permanent Teeth and the Ailments of Late Childhood .... 275
Dental Laws of North Dakota .... 295
About Platinum .... 301
Dangers of Hypnotism .... 303
Chronic Syphilitic Salivation .... 304
Plastic Surgery .... 304
Chicago Dental Society .... 305
Another Warning to Landlords .... 306

PROCEEDINGS.

Annual Meeting of the Northern Ohio Dental Association .... 308
International Congress .... 309

EDITORIAL.

American Medical Association .... 311
Americ n Dental Association .... 312
Association of Dental College Faculties .... 313
National Association of Dental Examiners .... 313
Georgia State Dental Society .... 314
Obituary .... 315

